# MFG‐E8 Accelerates Abdominal Aortic Aneurysm Formation by Enhancing ERK MAPK/NOX4 Axis‐Associated Oxidative Stress

**DOI:** 10.1111/cpr.70104

**Published:** 2025-07-24

**Authors:** Jie Xiao, Hai Hu, Minghui Zou, Chenhao Li, Dawei Deng, Xing Chen, Jinping Liu

**Affiliations:** ^1^ Department of Cardiovascular Surgery Zhongnan Hospital of Wuhan University Wuhan China; ^2^ Department of Critical Care Medicine, Union Hospital, Tongji Medical College Huazhong University of Science and Technology Wuhan China; ^3^ Hubei Provincial Engineering Research Center of Minimally Invasive Cardiovascular Surgery Wuhan China; ^4^ Wuhan Clinical Research Center for Minimally Invasive Treatment of Structural Heart Disease Wuhan China

## Abstract

MFG‐E8 promotes oxidative stress by upregulating NOX4 and activating the MAPK pathway, which increases ROS production and affects vascular smooth muscle cell (VSMC) apoptosis, thereby driving the progression of abdominal aortic aneurysm (AAA). Resveratrol can inhibit the expression and function of MFG‐E8, reduce ROS generation, and lower the incidence and severity of AAA, making it a potential therapeutic agent for AAA.

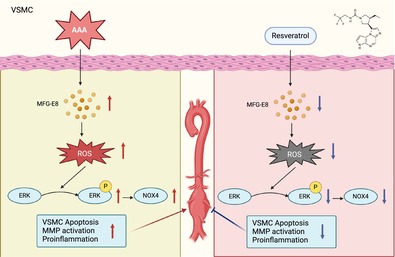

To the Editor,

Abdominal aortic aneurysm (AAA), a lethal vascular disease prevalent in elderly men, lacks effective pharmacological therapies as 90% of diagnosed cases are small (3.0–5.5 cm), with surgery reserved for diameters > 5.5 cm but carrying high risks [1, 2]. Pathogenesis involves endothelial dysfunction, vascular smooth muscle cell (VSMC) loss, and extracellular matrix degradation, with VSMCs critical for vascular integrity and emerging as key therapeutic targets [3, 4, 5, 6]. Milk fat globule‐epidermal growth factor VIII (MFG‐E8), a glycoprotein with dual pro‐ and anti‐inflammatory roles, regulates VSMC phenotypes in vascular diseases like atherosclerosis [7]. While previously linked to anti‐inflammatory effects in conditions like acute pancreatitis, recent studies highlight its pro‐inflammatory actions in aging, hypertension, and vascular injury, driving intima‐media thickening and pathological remodelling [8, 9, 10]. In vitro, MFG‐E8 modulates VSMC senescence [7, 11], inflammation [12], apoptosis [8], and matrix deposition [10], yet its role in AAA remains unstudied. This study uses MFG‐E8 wild‐type (WT) and knockout (KO) mice with angiotensin II (Ang II)‐induced AAA models to investigate whether MFG‐E8 promotes AAA formation. Mechanistic analyses focus on its impact on VSMC oxidative stress, aiming to clarify MFG‐E8's role in AAA pathogenesis and identify novel intervention targets for this deadly disease.

To investigate the role of MFG‐E8 in AAA and its potential as a therapeutic target, this study analysed human aortic tissues and used genetically modified mice and VSMCs. Human AAA tissues exhibited significantly higher MFG‐E8 expression at both protein (*p* = 0.0029, Figure 1A) and mRNA (*p* = 0.0241, Figure 1B) levels compared to normal aortic tissues, confirmed by immunofluorescence (*p* = 0.0023, Figure 1C), indicating its involvement in AAA pathogenesis.

**FIGURE 1:**
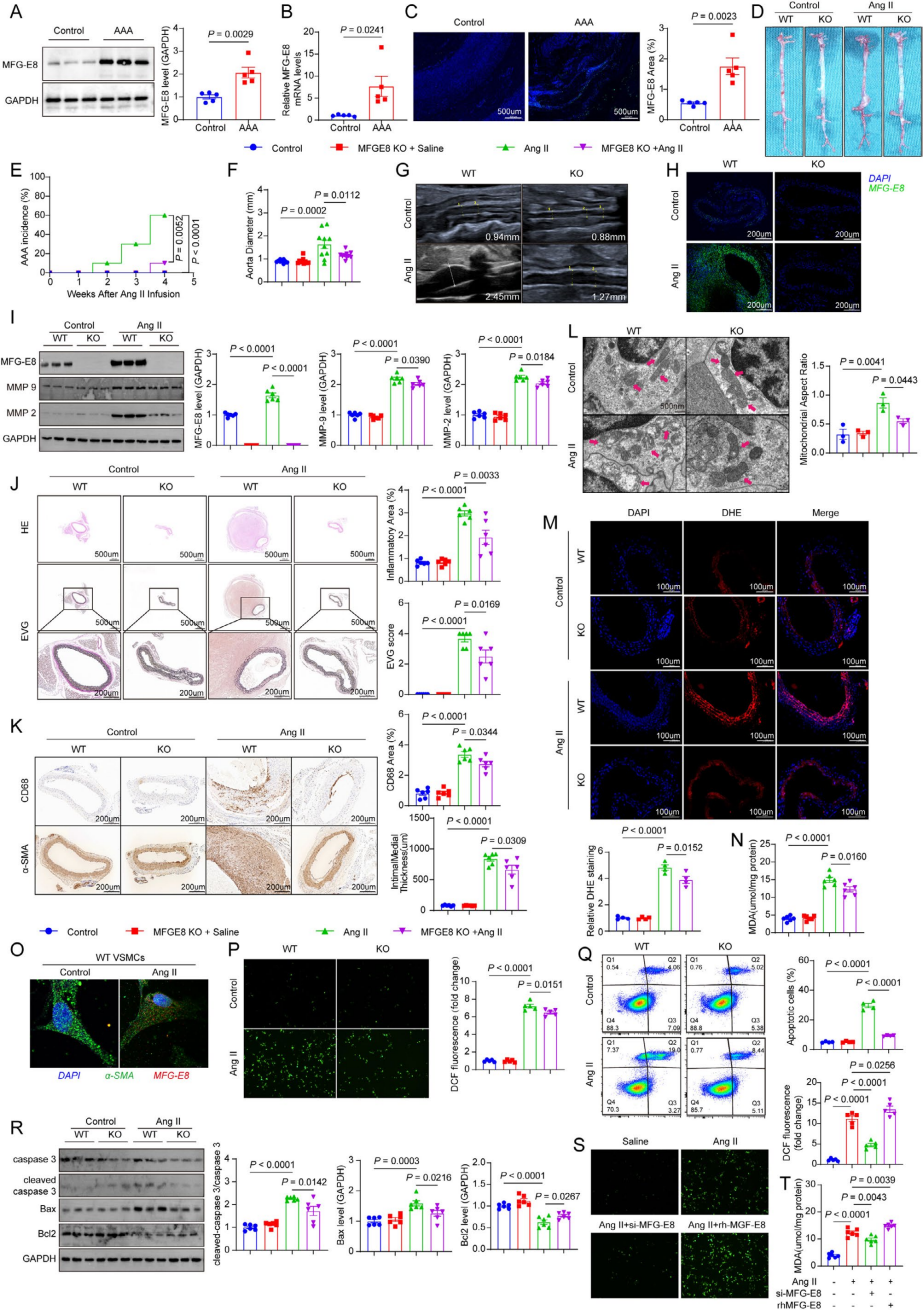
Elevated MFG‐E8 expression promotes abdominal aortic aneurysm formation by modulating ROS production and VSMC apoptosis. (A) MFG‐E8 expression in human abdominal aortic aneurysm specimens and normal abdominal aortic tissues was detected by Western blot. (B) MFG‐E8 mRNA levels were assessed in human samples. (C) MFG‐E8 expression in abdominal aortic aneurysm and normal abdominal aortic tissues was examined using immunofluorescence. (D) Typical gross images of abdominal aortic aneurysm modelling in each mouse group. (E) Comparison of aneurysm incidence among different mouse groups. (F) Comparison of abdominal aortic diameter in each mouse group. (G) Typical ultrasound measurement images of abdominal aorta in each mouse group. (H) Immunofluorescence staining of MFG‐E8 in abdominal aortic tissues from each mouse group. (I) Expression levels of inflammatory factor proteins in abdominal aortic tissues from each mouse group. (J) HE and EVG staining analysis of abdominal aortic tissues from each mouse group. (K) Immunohistochemical analysis of CD68 and α‐SMA in abdominal aortic tissues from each mouse group. (L) Transmission electron microscopy (TEM) analysis of mitochondrial morphology in abdominal aortic tissues from each mouse group. (M, N) DHE staining and MDA analysis of abdominal aortic tissues from each mouse group. (O) Extraction of primary abdominal aortic smooth muscle cells from WT mouse and confocal analysis of MFG‐E8 expression after AngII stimulation. (P) Extraction of primary abdominal aortic smooth muscle cells from each mouse group and DCF staining to assess ROS levels. (Q) Extraction of primary smooth muscle cells from each mouse group for flow cytometry analysis to detect apoptosis. (R) Western blot analysis of apoptosis‐related protein levels in primary smooth muscle cells from each mouse group. (S) DCF staining to assess ROS expression levels in WT VSMCs after different stimulations. (T) MDA assay to evaluate ROS levels in WT VSMCs after different stimulations.

To investigate the functional impact of MFG‐E8 in vivo, we utilised a well‐characterised model of Ang II‐induced AAA in WT and MFG‐E8 KO mice (Figure 1D,G). After 28 days of Ang II infusion, KO mice exhibited a significantly lower incidence of AAA compared to WT mice (*p* = 0.0052, Figure 1E), a reduction in the maximum aortic diameter (*p* = 0.0112, Figure 1F), and confirmed by immunofluorescence staining of MFG‐E8 in the aortic tissues of each group (Figure 1H). Furthermore, KO mice exhibited decreased MMP2 (*p* = 0.0184, Figure 1I), MMP9 expression (*p* = 0.0390, Figure 1I), reduced inflammatory cell infiltration (*p* = 0.0033, Figure 1J), and less elastic fibre degradation (*p* = 0.0169, Figure 1J). Additionally, the CD68 and α‐SMA staining revealed that the Ang II treated KO group exhibited significantly reduced macrophage infiltration (*p* = 0.0344, Figure 1K) and a marked decrease in intimal medial thickness (*p* = 0.0309, Figure 1K) compared to the Ang II‐treated WT group. Those results show MFG‐E8 drives vascular remodelling and inflammation. Given the established role of oxidative stress in driving VSMC apoptosis and ECM degradation in AAA, we explored whether MFG‐E8 influences reactive oxygen species (ROS) production. Transmission electron microscopy of aortic tissues revealed severe mitochondrial fragmentation and cristae loss in WT mice after Ang II treatment, changes that were markedly less pronounced in KO mice (*p* = 0.0443, Figure 1L). Dihydroethidium (DHE) staining, a marker for superoxide anions, and malondialdehyde (MDA) assays, which measure lipid peroxidation, both showed significantly lower ROS levels in KO mice compared to the WT group (ROS, *p* = 0.0152 Figure 1M; MDA, *p* = 0.016; Figure 1N).

Since VSMCs are the primary cellular components of the aortic wall and a major source of ROS [13, 14], we initially isolated primary VSMCs from WT mice. To investigate the expression of MFG‐E8, we performed immunofluorescence analysis on VSMCs with and without Ang II stimulation. Confocal microscopy revealed a significant upregulation of MFG‐E8 expression in VSMCs following Ang II treatment (Figure 1O). Next, we evaluated whether MFG‐E8 affects ROS production in vitro. The results showed that the ROS levels significantly increased in the WT group but were markedly reduced in the KO group after Ang II stimulation (*p* = 0.0151, Figure 1P). Then we assessed the effect of MFG‐E8 on VSMCs survival using flow cytometry and WB analysis. After Ang II stimulation, the apoptosis rate of VSMCs was significantly increased in the WT group, whereas it was significantly mitigated in the KO group (*p* < 0.0001, Figure 1Q). The protein levels of Bax (*p* = 0.0216, Figure 1R), Bcl2 (*p* = 0.0267, Figure 1R), and cleaved‐caspase3 (*p* = 0.0142, Figure 1R) showed similar results. Additionally, the DCF staining and MDA levels were significantly reduced after MFG‐E8 siRNA treatment (*p* < 0.0001, Figure 1S; *p* = 0.0043, Figure 1T), while conversely increased after rhMFG‐E8 treatment (*p* = 0.0256, Figure 1S; *p* = 0.0039, Figure 1T). The protein levels of Bax, Bcl2, and cleaved‐caspase3 showed similar results (Figure 2A).

**FIGURE 2:**
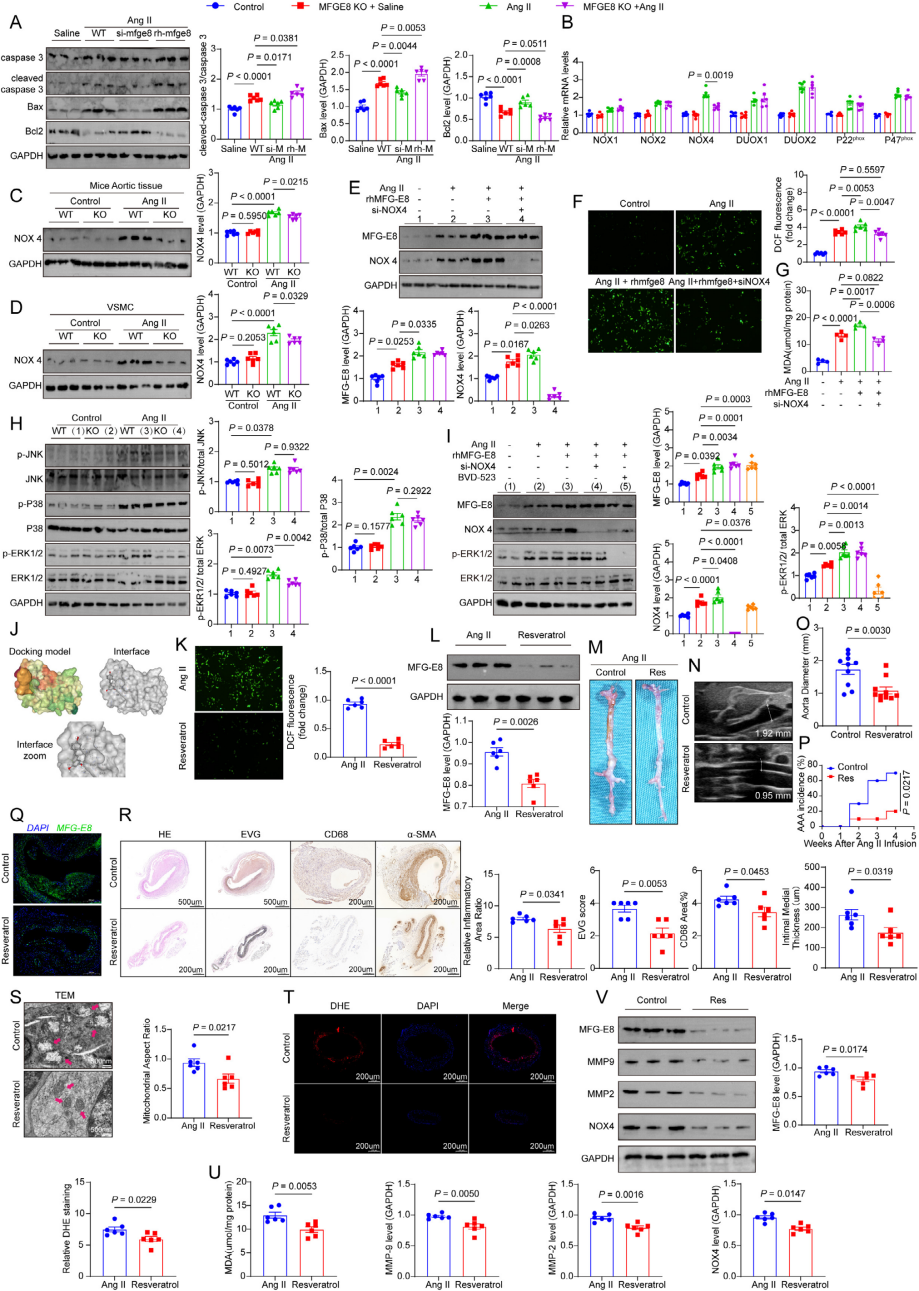
MFG‐E8 drives abdominal aortic aneurysm formation via NOX4/MAPK‐mediated oxidative stress: inhibition by resveratrol. (A) Protein level detection of apoptosis‐related protein expression in WT VSMCs after different stimulations. (B) mRNA analysis of NOX family‐related molecule expression in abdominal aortic tissues from each mouse group. (C) Western blot analysis of NOX4 expression in abdominal aortic tissues from each mouse group. (D) Extraction of primary abdominal aortic smooth muscle cells from each mouse group for Western blot detection of NOX4 expression. (E) Western blot analysis of MFG‐E8 and NOX4 expression in WT VSMCs after different stimulations. (F) DCF staining to assess ROS expression levels in WT VSMCs after different stimulations. (G) MDA assay to evaluate ROS levels in WT VSMCs after different stimulations. (H) Western blot analysis of JNK, P38, ERK, and their phosphorylated forms in abdominal aortic tissues from each mouse group. (I) Western blot analysis of MFG‐E8, NOX4, p‐ERK, and ERK expression levels in WT VSMCs after different stimulations. (J) Molecular docking technology to predict whether resveratrol could bind to MFG‐E8. (K) DCF staining to assess ROS expression levels in WT VSMCs after different stimulations. (L) Western blot analysis of MFG‐E8 expression in WT VSMCs after different stimulations. (M) Gross images of abdominal aorta from each mouse group after different stimulations. (N) Typical ultrasound measurement images of abdominal aorta from each mouse group. (O) Comparison of abdominal aortic diameter in each mouse group. (P) Comparison of aneurysm incidence among different mouse groups. (Q) Immunofluorescence staining results of MFG‐E8 in abdominal aortic tissues from each mouse group. (R) HE, EVG, CD68, and α‐SMA immunohistochemical staining images of abdominal aortic tissues from each mouse group. (S) Transmission electron microscopy (TEM) analysis of mitochondrial morphology in abdominal aortic tissues from each mouse group. (T, U) DHE staining and MDA analysis to assess ROS generation in abdominal aortic tissues from each mouse group. (V) Western blot analysis of MFG‐E8, MMP9, MMP2, and NOX4 expression levels in abdominal aortic tissues from each mouse group.

Current research indicates that the NOX family is the primary source of ROS in VSMCs, with NOX1, NOX2, and NOX4 highly expressed in these cells. Additionally, studies have demonstrated that the NOX family plays a critical role in the development and progression of AAA [15, 16, 17]. To investigate the underlying mechanisms by which MFG‐E8 induces oxidative stress, we first used qPCR to measure the mRNA expression levels of NOX family‐related molecules, including NOX1, NOX2, NOX4, DUOX1, DUOX2, P22^phox^, and P47^phox^, in the abdominal aortas of mice from different groups. We observed the significantly reduced expression of NOX4 in the Ang II + KO group compared to the Ang II + WT group (*p* = 0.0019, Figure 2B), and validated at the protein level (*p* < 0.001, Figure 2C). Then we isolated primary VSMCs from mice in each group and treated them with saline or Ang II. The results revealed that NOX4 protein levels were significantly decreased in the Ang II + KO group compared to the Ang II + WT group (*p* = 0.0329, Figure 2D). Furthermore, when the WT VSMCs were treated with si‐NOX4 and rhMFG‐E8 for 24 h, followed by Ang II stimulation for another 24 h, the expression of MFG‐E8 (*p* = 0.0335, Figure 2E) and NOX4 (*p* = 0.0263, Figure 2E) were elevated after rhMFG‐E8 treatment, while si‐NOX4 intervention significantly reduced NOX4 (*p* < 0.0001, Figure 2E) expression without affecting MFG‐E8 levels. These findings were validated by DCF (Figure 2F) and MDA (Figure 2G) staining. Mechanistically, previous studies have shown that ROS generation in AAA is primarily mediated through the MAPK signalling pathway, including JNK MAPK, P38 MAPK, and ERK MAPK. Therefore, we examined the expression of key molecules in the MAPK pathway, including phosphorylated JNK, phosphorylated P38, and phosphorylated ERK, in the abdominal aortas of mice from different groups [18]. The results indicated that Ang II treatment significantly activated all MAPK‐related molecules (Figure 2H). However, compared to the WT group, the KO group exhibited a significant reduction in p‐ERK levels, while total ERK levels remained unchanged (*p =* 0.0042, Figure 2H). To further confirm the role of MFG‐E8 in the ERK MAPK pathway, WT VSMCs were treated with rhMFG‐E8 for 24 h, followed by Ang II stimulation. The results showed that Ang II stimulation significantly increased p‐ERK levels (*p* = 0.0058, Figure 2I), and rhMFG‐E8 treatment further enhanced this effect (*p* = 0.0013, Figure 2I). These findings indicate that MFG‐E8 can promote ERK MAPK pathway activation. To explore the relationship between NOX4 and p‐ERK, cells were treated with si‐NOX4 (NOX4 siRNA) or the ERK pathway inhibitor BVD‐523, followed by Ang II stimulation. The results demonstrated that inhibition of the ERK pathway significantly reduced NOX4 expression levels (*p* = 0.0376, Figure 2I). These findings suggest that MFG‐E8 exacerbates oxidative stress by promoting NOX4 expression and activating the ERK MAPK pathway, thereby inducing VSMC apoptosis.

Resveratrol is a naturally occurring compound with various biological activities, including antioxidant, anti‐inflammatory, and antiproliferative effects [19, 20]. We first used molecular docking technology to predict whether resveratrol could bind to MFG‐E8; the results demonstrated that resveratrol is capable of forming a binding interaction with MFG‐E8 (Figure 2J). In our study, we found that the antioxidant compound resveratrol reduced MFG‐E8 expression and ROS levels in VSMCs (*p* < 0.0001, Figure 2K; *p* = 0.0026, Figure 2L). In Ang II‐induced mice, resveratrol decreased AAA incidence (Figure 2M,N; *p* = 0.0217, Figure 2P), aortic diameter (*p* = 0.0030, Figure 2O), and improved pathological features like inflammation and elastic fibre degradation (Figure 2Q,R). It alleviated mitochondrial damage (*p* = 0.0217, Figure 2S), alleviated the level of DHE (*p* = 0.0229, Figure 2T) and MDA (*p* = 0.0053, Figure 2U), reduced NOX4 (*p* = 0.0147, Figure 2V), MMP2 (*p* = 0.0016, Figure 2V), MMP9 (*p* = 0.0050, Figure 2V) expression, and suppressed MFG‐E8 levels (*p* = 0.0174, Figure 2V), indicating it targets MFG‐E8 to mitigate oxidative stress and AAA progression.

In summary, our study through in vivo and in vitro experiments first explored the role of MFG‐E8 in AAA development, revealing its critical role in AAA progression associated with pathological features like vascular smooth muscle cell apoptosis, extracellular matrix degradation, and intensified inflammation. Mechanistically, MFG‐E8 regulates ROS production by activating the ERK MAPK pathway to upregulate NOX4 expression. Resveratrol, by downregulating MFG‐E8, significantly mitigates these pathological changes, reducing oxidative stress and improving AAA‐related vascular remodelling. These findings highlight MFG‐E8 as a pivotal driver of AAA pathogenesis through the ERK MAPK/NOX4/ROS axis and propose it as a potential therapeutic target. While animal models effectively modelled AAA, clinical translation requires validation. Resveratrol's efficacy offers a promising direction for developing pharmacological interventions, though its long‐term safety and efficacy in humans need further study. This research bridges molecular mechanisms and potential clinical applications, addressing an unmet need in AAA treatment.

## Author Contributions

Jie Xiao and Xing Chen designed and performed the experiments, analysed the data, and wrote the manuscript. Jinping Liu and Xing Chen reviewed the manuscript. Jie Xiao, Hai Hu, Chenhao Li and Dawei Deng performed the experiment. Jie Xiao, Xing Chen and Hai Hu analysed the data. All authors contributed to the article and approved the submitted version.

## Ethics Statement

The experimental animals used in this study were approved by the Experimental Animal Welfare Ethics Committee of Zhongnan Hospital of Wuhan University (Approval No. ZN2023102) and complied with the principles of the Declaration of Helsinki and relevant local laws and regulations. All animal experimental procedures were strictly conducted in accordance with the Guide for the Care and Use of Laboratory Animals. Additionally, the human samples used in this study were approved by the Medical Ethics Committee of Zhongnan Hospital of Wuhan University (Approval No. 2023187), in compliance with the principles of the Chinese NMPA/GCP, the Declaration of Helsinki, and relevant laws and regulations.

## Conflicts of Interest

The authors declare no conflicts of interest.

## Data Availability

The data that support the findings of this study are available from the corresponding author upon reasonable request.

## References

[1] N. Sakalihasan , J. B. Michel , A. Katsargyris , et al., “Abdominal Aortic Aneurysms,” Nature Reviews. Disease Primers 4 (2018): 34. 10.1038/s41572-018-0030-7 30337540

[2] J. Golledge , “Abdominal Aortic Aneurysm: Update on Pathogenesis and Medical Treatments,” Nature Reviews. Cardiology 16 (2019): 225–242. 30443031 10.1038/s41569-018-0114-9

[3] J. Gao , H. Cao , G. Hu , et al., “The Mechanism and Therapy of Aortic Aneurysms,” Signal Transduction and Targeted Therapy 8 (2023): 55. 36737432 10.1038/s41392-023-01325-7 PMC9898314

[4] S. Jana , M. Hu , M. Shen , and Z. Kassiri , “Extracellular Matrix, Regional Heterogeneity of the Aorta, and Aortic Aneurysm,” Experimental & Molecular Medicine 51 (2019): 1–15. 10.1038/s12276-019-0286-3 PMC6923362 31857579

[5] H. Sawada , D. L. Rateri , J. J. Moorleghen , M. W. Majesky , and A. Daugherty , “Smooth Muscle Cells Derived From Second Heart Field and Cardiac Neural Crest Reside in Spatially Distinct Domains in the Media of the Ascending Aorta‐Brief Report,” Arteriosclerosis, Thrombosis, and Vascular Biology 37 (2017): 1722–1726. 28663257 10.1161/ATVBAHA.117.309599 PMC5570666

[6] R. A. Quintana and W. R. Taylor , “Cellular Mechanisms of Aortic Aneurysm Formation,” Circulation Research 124 (2019): 607–618. 30763207 10.1161/CIRCRESAHA.118.313187 PMC6383789

[7] H. Y. Chiang , P. H. Chu , S. C. Chen , et al., “MFG‐E8 Promotes Osteogenic Transdifferentiation of Smooth Muscle Cells and Vascular Calcification by Regulating TGF‐beta1 Signaling,” Communications Biology 5 (2022): 364. 35440618 10.1038/s42003-022-03313-z PMC9018696

[8] Y. Ren , W. Liu , L. Zhang , et al., “Milk Fat Globule EGF Factor 8 Restores Mitochondrial Function via Integrin‐Medicated Activation of the FAK‐STAT3 Signaling Pathway in Acute Pancreatitis,” Clinical and Translational Medicine 11 (2021): e295. 33634976 10.1002/ctm2.295 PMC7828261

[9] J. Ren , B. Zhu , G. Gu , et al., “Schwann Cell‐Derived Exosomes Containing MFG‐E8 Modify Macrophage/Microglial Polarization for Attenuating Inflammation via the SOCS3/STAT3 Pathway After Spinal Cord Injury,” Cell Death & Disease 14 (2023): 70. 36717543 10.1038/s41419-023-05607-4 PMC9887051

[10] B. Liu , B. Zhang , J. Qi , et al., “Targeting MFGE8 Secreted by Cancer‐Associated Fibroblasts Blocks Angiogenesis and Metastasis in Esophageal Squamous Cell Carcinoma,” Proceedings of the National Academy of Sciences of the United States of America 120 (2023): e2307914120. 37816055 10.1073/pnas.2307914120 PMC10589644

[11] L. Ni , L. Liu , W. Zhu , et al., “Inflammatory Role of Milk Fat Globule‐Epidermal Growth Factor VIII in Age‐Associated Arterial Remodeling,” Journal of the American Heart Association 11 (2022): e022574. 36000422 10.1161/JAHA.121.022574 PMC9496444

[12] L. Zhang , R. Tian , X. Yao , et al., “Milk Fat Globule‐Epidermal Growth Factor‐Factor 8 Improves Hepatic Steatosis and Inflammation,” Hepatology 73 (2021): 586–605. 32297339 10.1002/hep.31277

[13] J. Cheng , R. Zhang , C. Li , et al., “A Targeting Nanotherapy for Abdominal Aortic Aneurysms,” Journal of the American College of Cardiology 72 (2018): 2591–2605. 30466517 10.1016/j.jacc.2018.08.2188

[14] J. Li , X. Li , S. Song , et al., “Mitochondria Spatially and Temporally Modulate VSMC Phenotypes via Interacting With Cytoskeleton in Cardiovascular Diseases,” Redox Biology 64 (2023): 102778. 37321061 10.1016/j.redox.2023.102778 PMC10277590

[15] K. L. Siu , Q. Li , Y. Zhang , et al., “NOX Isoforms in the Development of Abdominal Aortic Aneurysm,” Redox Biology 11 (2017): 118–125. 27912196 10.1016/j.redox.2016.11.002 PMC5133668

[16] S. J. Forrester , G. W. Booz , C. D. Sigmund , et al., “Angiotensin II Signal Transduction: An Update on Mechanisms of Physiology and Pathophysiology,” Physiological Reviews 98 (2018): 1627–1738. 29873596 10.1152/physrev.00038.2017 PMC6335102

[17] Y. Zhang , P. Murugesan , K. Huang , and H. Cai , “NADPH Oxidases and Oxidase Crosstalk in Cardiovascular Diseases: Novel Therapeutic Targets,” Nature Reviews. Cardiology 17 (2020): 170–194. 31591535 10.1038/s41569-019-0260-8 PMC7880919

[18] C. Xu , J. Xu , C. Zou , et al., “Chronic Intermittent Hypoxia Regulates CaMKII‐Dependent MAPK Signaling to Promote the Initiation of Abdominal Aortic Aneurysm,” Oxidative Medicine and Cellular Longevity 2021 (2021): 2502324. 34970414 10.1155/2021/2502324 PMC8714336

[19] S. Jin and P. M. Kang , “A Systematic Review on Advances in Management of Oxidative Stress‐Associated Cardiovascular Diseases,” Antioxidants (Basel) 13 (2024): 13. 10.3390/antiox13080923 PMC11351257 39199169

[20] S. Zheng , P. S. Tsao , and C. Pan , “Abdominal Aortic Aneurysm and Cardiometabolic Traits Share Strong Genetic Susceptibility to Lipid Metabolism and Inflammation,” Nature Communications 15 (2024): 5652. 10.1038/s41467-024-49921-7 PMC11226445 38969659

